# Phylogeography of *Borrelia* spirochetes in *Ixodes pacificus* and *Ixodes spinipalpis* ticks highlights differential acarological risk of tick-borne disease transmission in northern versus southern California

**DOI:** 10.1371/journal.pone.0214726

**Published:** 2019-04-04

**Authors:** Ian Rose, Melissa Hardstone Yoshimizu, Denise L. Bonilla, Natalia Fedorova, Robert S. Lane, Kerry A. Padgett

**Affiliations:** 1 California Department of Public Health, Vector-Borne Disease Section, Richmond, CA, United States of America; 2 Department of Environmental Science Policy and Management, University of California, Berkeley, CA, United States of America; University of Kentucky College of Medicine, UNITED STATES

## Abstract

The common human-biting tick, *Ixodes pacificus*, is the primary vector of the Lyme disease spirochete, *Borrelia burgdorferi* sensu stricto (ss) in western North America and has been found to harbor other closely-related spirochetes in the *Borrelia burgdorferi* sensu lato (sl) complex. Between 2008–2015, 11,066 adult and 3,815 nymphal *I*. *pacificus* and five adult and 144 nymphal *Ixodes spinpalpis*, a commonly collected wildlife tick, were collected from 42 California counties. *Borrelia burgdorferi* sl was detected in 1.2% and 3.8% *I*. *pacificus* adults and nymphs, respectively. Results from this study indicate genetic diversity and geographic structure of *B*. *burgdorferi* sl in California *I*. *pacificus* ticks, by sequence comparison of the16S rRNA gene, with *B*. *burgdorferi* ss, the agent of Lyme disease, found only in *I*. *pacificus* collected from the north and central coastal and Sierra Nevada foothill regions; *B*. *burgdorferi* ss was not detected in ticks tested from southern California. In contrast, *Borrelia bissettiae*, a member of the *B*. *burgdorferi* sl complex, was detected in both *I*. *pacificus* and *I*. *spinipalpis*, in the coastal region of both northern and southern California, but was absent from ticks in the Sierra Nevada foothills. In a similar pattern to *B*. *bissettiae*, *Borrelia americana* (a member of the *B*. *burgdorferi* sl complex) was detected in a single adult *I*. *pacificus* from the north coast and two *I*. *spinipalpis* nymphs from south-coastal California. This study highlights that the geographic area of Lyme disease acarological risk in California is the north-central and Sierra Nevada foothill regions of the state with little to no risk in the southern regions of the state.

## Introduction

There is considerable diversity in the *Borrelia burgdorferi* sensu lato (sl) complex. Worldwide, members of the *B*. *burgdorferi* sl complex include at least 22 named genospecies worldwide, of which ten genospecies have been identified to date in North America: *Borrelia burgdorferi* sensu stricto (ss), *B*. *americana*, *B*. *andersoni*, *B*. *bissettiae*, *B*. *californiensis*, *B*. *carolinensis*, *B*. *garinii*, *B*. *kurtenbachii*, *B*. *laneii*, *and B*. *mayonii* [1–4]. In California, only *B*. *burgdorferi* ss, *B*. *americana*, *B*. *bissettiae*, *B*. *californiensis*, and *B*. *lanei* have been described in *Ixodes pacificus* ticks, along with currently-uncharacterized *Borrelia* species [2, 5, 6]. While *B*. *burgdorferi* ss is the primary etiologic agent of Lyme disease in North America, and *B*. *mayonii* causes Lyme disease in the upper Midwest, recent studies suggest that *B*. *bissettiae*, a known human pathogen in Europe [7–9], may also infect people in the southeastern United States [10] and California [11].

The recognition of variation within the *B*. *burgdorferi* sl complex has direct public health implications. By considering all *B*. *burgdorferi* sl positive ticks “positive,” the prevalence for infection, and thus the acarological index of Lyme disease risk, has been overestimated [12]. Furthermore, genospecies other than *B*. *burgdorferi* ss may cause human disease, which could manifest with potentially different etiologies.

In this study, molecular methods were used to characterize the *Borrelia* genospecies of *B*. *burgdorferi* sl-positive *I*. *pacificus* and *I*. *spinipalpis* to investigate large-scale spatial patterns of *Borrelia* genospecies present in California. Previous studies have identified *B*. *burgdorferi* sl and *B*. *miyamotoi* in California *I*. *pacificus* but did not further resolve *B*. *burgdorferi* to genospecies [12]. *Ixodes pacificus* is a common human-biting tick found along the Pacific Coast of the United States and is the primary vector of Lyme disease to people in this region (https://www.cdc.gov/ticks/geographic_distribution.html). These data enabled us to clarify the relative acarological risk of human exposure to pathogenic *Borrelia* in a heavily populated state in western North America.

## Materials and methods

### Tick collection

The California Department of Public Health (CDPH), Vector-Borne Disease Section performs routine surveillance and testing of sylvatic *Ixodes* ticks for *Borrelia* spp. spirochetes. This includes relapsing fever group *Borrelia* (e.g., *B*. *miyamotoi*) and members of the *B*. *burgdoferi* sl complex [12]. *Ixodes pacificus* adults and nymphs were collected throughout the state of California from 2008 to 2015. Ticks were collected from low vegetation, leaf litter, or other substrates (e.g., rocks or downed logs) by CDPH and county public health agencies using 1-m^2^ white double nap flannel “flag” attached to a 1.5-m wooden dowel. In most instances, ticks were collected from public lands such as regional or state parks. Ticks were collected all months of the year, with adult ticks most commonly collected in the winter months and the nymphs in the spring and summer months. Adult and nymphal *I*. *spinipalpis* ticks were collected opportunistically by flagging, during the same collection events; this tick species rarely attaches to people but parasitizes wildlife such as woodrats (*Neotoma fuscipes* and *N*. *macrotis*) and may be an important bridge vector for *B*. *burgdorferi* sl. Ticks were either maintained alive within 37-mL polystyrene containers (Fisher Scientific, USA) in sealed plastic bags with moistened paper toweling at 3°C or retained in 70% ethanol within 1.5-mL microcentrifuge snap-cap tubes.

### Tick preparation

Ticks were tested individually by direct florescence antibody assay (DFA), using *Borrelia* generic fluorescent-labeled antibodies to detect *Borrelia* species spirochetes. Live ticks were dissected onto etched microscope slides and stained with FITC-labeled BacTrace Anti-*Borrelia* Species Antibody (KPA) [13, 14]. Half of each dissected tick was transferred to a 2-mL snap cap tube that contained 20ul sterile PBS and stored at -80°C for later use. At least 100 visual fields were examined at 400X magnification for the presence of *Borrelia* spirochetes.

Ticks that tested positive for *Borrelia* spirochetes by DFA were further analyzed to determine the *Borrelia* genospecies. DNA from frozen tick tissues was extracted using QIAGEN DNeasy Blood and Tissue Kit (Hercules, CA) according to manufacturer’s instructions.

### Molecular analyses

DNA from DFA-positive ticks was screened for *B*. *miyamotoi* and *B*. *burgdorferi* sl using a TaqMan assay [15]. Forward and reverse primers were, respectively, 5’GCTGTAAACGATGCACACTTGGT3’ and 5′GGCGGCACACTTAACACGTTAG 3’ targeting a 1130bp 16S rRNA sequence as described [15]. The probes used were 6FAM-TTCGGTACTA ACTTTTAGTTA corresponding to *B*. *burgdorferi* sl and VIC-CGGTACTAACCTTTCGAT TA corresponding to *B*. *miyamotoi* with the 3’ ends modified with a minor groove binding protein. All reactions were performed in a final volume of 25 ul on a BioRad CFX96 Real-Time Detection System containing 2x SooFast Probes SuperMix (BioRad), primers (900 nM), and probes (200 nM) per reaction. Thermal cycling conditions were as follows: 95°C for 2 min, 45 cycles of 95°C for 5 sec, and 63°C for 30 sec.

A 1130bp section of the 16S rRNA gene was amplified from TaqMan positive *B*. *burgdorferi* sl ticks. Forward and reverse primers were, respectively 5’CTGGCAGTGCGTCTTAAGCA3’ [16] and 5’GACTTATCACCGGCAGTCTTA3’ [17]. PCRs were performed in 25ul volumes with final concentrations of 0.2uM for forward and reverse primers, 200uM dNTPs, and 0.625 units of Taq DNA polymerase per reaction. Thermal cycling conditions were: 94°C for 1 min, 45 cycles of 94°C for 1 min, 61.2°C for 30 sec, and 72°C for 90 sec, followed by final extension of 72°C for 7 min. The PCR products were visualized on a 2% Life Technologies E-gel stained with SYBRgreen (Carlsbad, CA).

PCR product was purified using either Affymatrix ExoSAP-IT (Santa Clara, CA) or QIAquick PCR Purification Kit, according to manufacturer’s instructions, respectively. Samples were sequenced by Quintara (http://www.quintarabio.com/). For each sample, forward and reverse sequences were obtained. The forward and reverse reads were aligned using ClustalOmega (http://www.ebi.ac.uk/Tools/msa/clustalo/) and edited manually. Electropherograms were examined for the presence of conflicting base calls using ApE (http://biologylabs.utah.edu/jorgensen/wayned/ape/) to address the possibility that a tick was co-infected with more than one genospecies of *B*. *burgdorferi* sl. In instances where a sample seemed to produce more than one PCR product, suggesting multiple *B*. *burgdorferi* sl infections, PCR products were cloned using a Qiagen PCR Cloning Kit. Inserts from 3–5 colonies were then Sanger sequenced as described above.

The acquired 16S rRNA sequences were aligned with 16S rRNA sequences from other *Borrelia* genospecies retrieved from GenBank. Sequences were aligned using ClustalOmega (http://www.ebi.ac.uk/Tools/msa/clustalo/). The 16S rRNA sequence from *B*. *miyamotoi* (Genbank accession number AB904793.1) served as the outgroup. After manual refinement, conserved regions were identified using the Gblocks feature of the Phylogeny.fr suite [18, 19]. The HKY+G nucleotide substitution model was selected using TOPALiv2’s model selection feature [20]. TOPALi was then used to launch MrBayes to construct a phylogenetic tree [21–23]. The tree was generated using two runs of 9,000,000 generations with 35% burn in and trees sampled every 1000 generations.

## Results

In total, 11,066 *I*. *pacificus* adults, collected from 2008 to 2015, were screened for *Borrelia* spp. by DFA. Of these, 228 adults (2.1%) were DFA positive for *Borrelia* spirochetes and, of these, 128 (1.2%) were *B*. *burgdorferi* sl positive and 96 (0.9%) were *B*. *miyamotoi* positive when tested by TaqMan assay; four positive ticks were not able to amplify. A subset of 27 of the *B*. *burgdorferi* sl-positive adult ticks were characterized to genospecies by sequence comparison. This subset of positive ticks was selected to optimize the number of ticks tested from different regions of the state. *Borrelia burgdorferi* ss was detected in 11 counties, *B*. *bissettiae* was detected in two counties, and *B*. *americana* was detected from one county (Table 1).

**Table 1:** Adult and nymphal *Ixodes pacificus* and *Ixodes spinipalpis* collected in California and tested for *Borrelia* spp., 2008–2015.

Region	County	Tick species	Life stage	# tested	# samples positive for *B*. *burgdorferi* s.l.	% prevalence of *B*. *burgdorferi* s.l.	# in subset with *B*. *burgdorferi* s.l. genomospecies determined ^a^	*B*. *americana*	*B*. *bissettiae*	*B*. *burgdorferi ss*
**Central Coast**										
	Monterey	*Ixodes pacificus*	Adult	140	0	0.0				
		*Ixodes pacificus*	Nymphs	35	0	0.0				
	San Benito	*Ixodes pacificus*	Adult	47	0	0.0				
		*Ixodes pacificus*	Nymphs	4	0	0.0				
	San Luis Obispo	*Ixodes pacificus*	Adult	298	1	0.3	1			1
		*Ixodes pacificus*	Nymphs	0	0					
**Central Valley**										
	Colusa	*Ixodes pacificus*	Adult	13	0	0.0				
		*Ixodes pacificus*	Nymphs	0	0					
	Glenn	*Ixodes pacificus*	Adult	171	2	1.2				
		*Ixodes pacificus*	Nymphs	0	0					
	Kern	*Ixodes pacificus*	Adult	57	0	0.0				
		*Ixodes pacificus*	Nymphs	0	0					
	Sacramento	*Ixodes pacificus*	Adult	88	3	3.4				
		*Ixodes pacificus*	Nymphs	190	18	9.5	1			1
		*Ixodes spinipalpis*	Nymphs	11	1	9.1				
	San Joaquin	*Ixodes pacificus*	Adult	13	0	0.0				
		*Ixodes pacificus*	Nymphs	3	0	0.0				
	Stanislaus	*Ixodes pacificus*	Adult	211	0	0.0				
		*Ixodes pacificus*	Nymphs	0	0					
	Yuba	*Ixodes pacificus*	Adult	424	7	1.7				
		*Ixodes pacificus*	Nymphs	30	0	0.0				
**North Coastal**										
	Alameda	*Ixodes pacificus*	Adult	466	5	1.1				
		*Ixodes pacificus*	Nymphs	29	0	0.0				
	Contra Costa	*Ixodes pacificus*	Adult	285	0	0.0				
		*Ixodes pacificus*	Nymphs	264	4	1.5	1			1
		*Ixodes spinipalpis*	Nymphs	1	0	0.0				
	Humboldt	*Ixodes pacificus*	Adult	30	0	0.0				
		*Ixodes pacificus*	Nymphs	2	0	0.0				
	Lake	*Ixodes pacificus*	Adult	253	2	0.8	2			2
		*Ixodes pacificus*	Nymphs	492	14	2.8	7			7
	Marin	*Ixodes pacificus*	Adult	682	14	2.1	4		1	3
		*Ixodes pacificus*	Nymphs	331	24	7.3	10			10
	Mendocino	*Ixodes pacificus*	Adult	61	0	0.0				
		*Ixodes pacificus*	Nymphs	19	0	0.0				
	Napa	*Ixodes pacificus*	Adult	385	3	0.8				
		*Ixodes pacificus*	Nymphs	342	3	0.9				
	San Mateo	*Ixodes pacificus*	Adult	620	15	2.4	6	1	2	3
		*Ixodes spinipalpis*	Adult	1	0	0.0				
		*Ixodes pacificus*	Nymphs	96	4	4.2				
		*Ixodes spinipalpis*	Nymphs	5	1	20.0				
	Santa Clara	*Ixodes pacificus*	Adult	182	3	1.6	3			3
		*Ixodes pacificus*	Nymphs	134	9	6.7	6			6
	Santa Cruz	*Ixodes pacificus*	Adult	893	3	0.3	1			1
		*Ixodes pacificus*	Nymphs	476	16	3.4	6		1	5
		*Ixodes spinipalpis*	Nymphs	4	1	25.0				
	Solano	*Ixodes pacificus*	Adult	121	0	0.0				
		*Ixodes pacificus*	Nymphs	0	0					
	Sonoma	*Ixodes pacificus*	Adult	216	1	0.5				
		*Ixodes pacificus*	Nymphs	337	3	0.9	1			1
	Trinity	*Ixodes pacificus*	Adult	56	0	0.0				
		*Ixodes pacificus*	Nymphs	0	0					
**Sierra-Nevada Foothills**										
	Amador	*Ixodes pacificus*	Adult	256	3	1.2				
		*Ixodes pacificus*	Nymphs	116	7	6.0				
	Butte	*Ixodes pacificus*	Adult	441	8	1.8	1			1
		*Ixodes pacificus*	Nymphs	309	17	5.5	3			3
	Calaveras	*Ixodes pacificus*	Adult	537	7	1.3	1			1
		*Ixodes pacificus*	Nymphs	30	0	0.0				
	El Dorado	*Ixodes pacificus*	Adult	312	10	3.2	5			5
		*Ixodes pacificus*	Nymphs	233	21	9.0	2			2
		*Ixodes spinipalpis*	Nymphs	12	3	25.0				
	Inyo	*Ixodes pacificus*	Adult	2	0	0.0				
		*Ixodes pacificus*	Nymphs	0	0					
	Mariposa	*Ixodes pacificus*	Adult	181	1	0.6	1			1
		*Ixodes pacificus*	Nymphs	15	0	0.0				
	Nevada	*Ixodes pacificus*	Adult	551	7	1.3	1			1
		*Ixodes pacificus*	Nymphs	173	6	3.5				
	Placer	*Ixodes pacificus*	Adult	10	1	10.0				
		*Ixodes pacificus*	Nymphs	40	0	0.0				
	Shasta	*Ixodes pacificus*	Adult	460	26	5.7				
		*Ixodes pacificus*	Nymphs	90	0	0.0				
	Sierra	*Ixodes pacificus*	Adult	28	0	0.0				
		*Ixodes pacificus*	Nymphs	0	0					
	Siskiyou	*Ixodes pacificus*	Adult	13	0	0.0				
		*Ixodes pacificus*	Nymphs	0	0					
	Tuolumne	*Ixodes pacificus*	Adult	64	0	0.0				
		*Ixodes pacificus*	Nymphs	3	0	0.0				
**Southern Region**										
	Los Angeles	*Ixodes pacificus*	Adult	360	0	0.0				
		*Ixodes pacificus*	Nymphs	11	0	0.0				
	Orange	*Ixodes pacificus*	Adult	659	2	0.3				
		*Ixodes spinipalpis*	Adult	4	1	25.0	1		1	
		*Ixodes pacificus*	Nymphs	0	0					
		*Ixodes spinipalpis*	Nymphs	111	20	18.0	8	2	6	
	Riverside	*Ixodes pacificus*	Adult	180	0	0.0				
		*Ixodes pacificus*	Nymphs	1	0	0.0				
	San Bernardino	*Ixodes pacificus*	Adult	678	3	0.4				
		*Ixodes pacificus*	Nymphs	1	0	0.0				
	San Diego	*Ixodes pacificus*	Adult	58	0	0.0				
		*Ixodes pacificus*	Nymphs	0	0					
	Santa Barbara	*Ixodes pacificus*	Adult	496	1	0.2				
		*Ixodes pacificus*	Nymphs	9	0	0.0				
	Ventura	*Ixodes pacificus*	Adult	68	0	0.0				
		*Ixodes pacificus*	Nymphs	0	0					

^a^ Due to screening a subset of samples positive for *B*. *burgdorferi* s.l., a true prevalence of genospecies by county could not be determined.

Similarly, 204 of the 3,815 nymphal *I*. *pacificus* were positive for *Borrelia* spp. by DFA. Of these, 146 (3.8%) of DFA-positive nymphs tested positive for *B*. *burgdorferi* sl and 52 (1.4%) tested positive for *B*. *miyamotoi* by TaqMan assay; six positive ticks were not able to amplify. Of the 37 *Borrelia-*positive *I*. *pacificus* nymphs that were genotyped, 36 were positive for *B*. *burgdorferi* ss from nine counties, and one was positive for *B*. *bissettiae* (Table 1).

Five *I*. *spinipalpis* adult ticks were collected from two counties; a single female from Orange County was positive for *B*. *bissettiae* (Table 1). In addition, 144 *I*. *spinipalpis* nymphs were tested from six counties. Of these 26 (18.1%) *I*. *spinipalpis* nymphs that were *B*. *burgdorferi* sl positive, two (1.4%) were *B*. *americana* positive, and six (4.2%) were *B*. *bissettiae* positive (Table 1). None of the *I*. *spinipalpis* adults or nymphs tested positive for either *B*. *burgdorferi* ss or *B*. *miyamotoi*.

**Fig 1:**
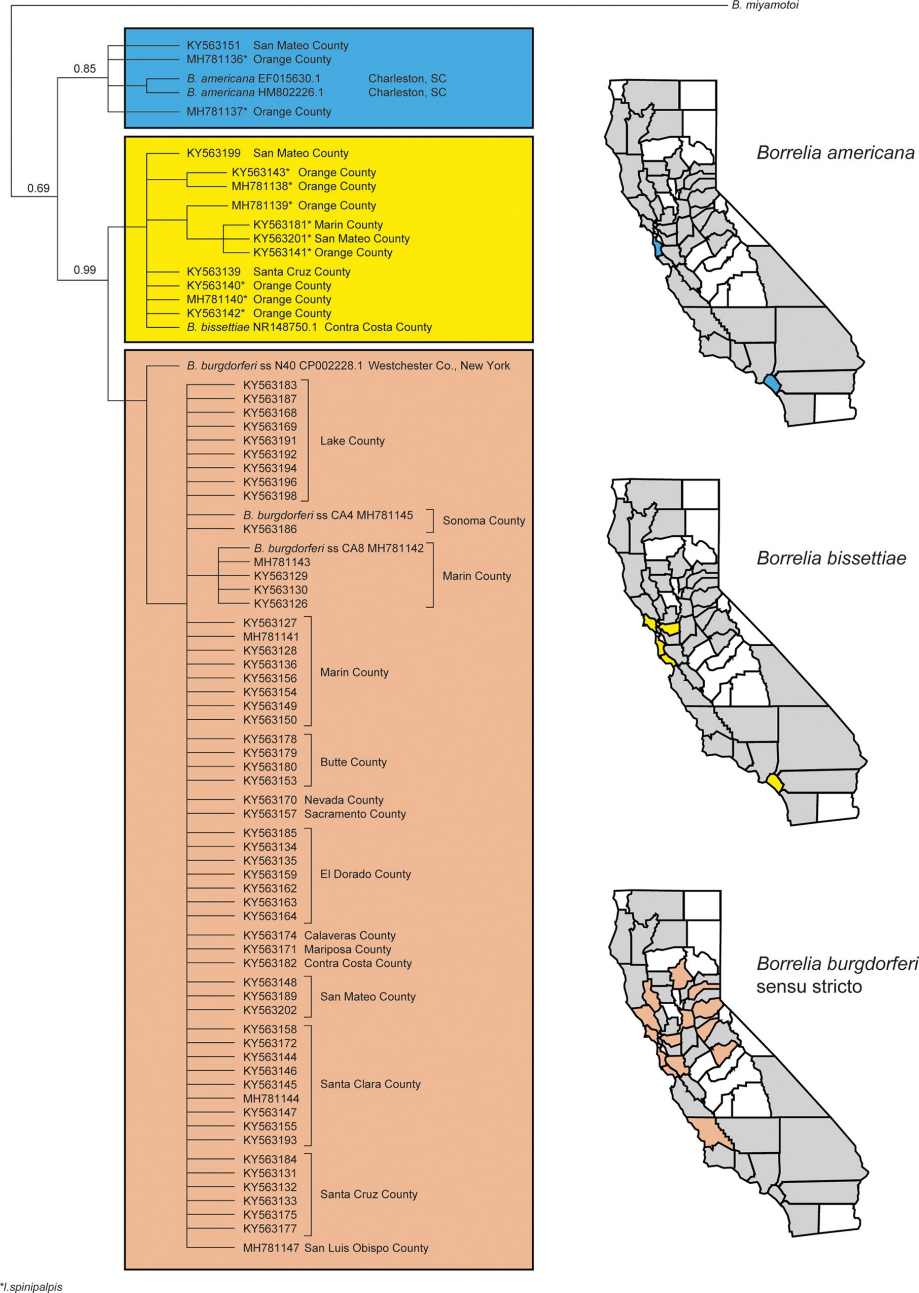
*Borrelia* genospecies detected in *Ixodes pacificus* and *Ixodes spinipalpis* ticks in California counties, 2008–2015.

The 16S rRNA sequence fragments obtained from sequencing a subset of positive amplicons (27 from adult ticks, 45 from nymphal ticks) were used to construct a phylogenetic tree (Table 2). *Borrelia* spp. from *Ixodes* ticks clustered into three clades, each containing a sequence from a GenBank-obtained *Borrelia* genospecies (Fig 1). The clade that contained *B*. *burgdorferi* ss was the largest with 58 samples and two controls (CA4 and CA8). The clade that included a *B*. *bissettiae* control (CA389) also included 11 tick-derived samples from along the northern and southern coastal regions of the state. In northern California, *B*. *bissettiae* was detected in *I*. *pacificus* whereas in southern California, *B*. *bissettiae* was detected in *I*.*spinipalpis* only. The *B*. *americana* clade included two positive *I*. *spinipalpis* nymphs from Orange County in southern California, one positive *I*. *pacificus* adult from the north-coastal county of San Mateo, and GenBank-derived sequence controls from Charleston County South Carolina (accession numbers HM802226, EU081286) (Table 2). Branch lengths are non-informative.

**Table 2:** Summary of California *Borrelia* positive ticks.

Borrelia spp.	County	Tick species	Life stage/sex	Tick no.	GenBank Accession No.	Latitude, Longitude
*Borrelia americana*	Orange	*I*. *spinipalpis*	Nymph	12–0751	MH781136	33.573899, -117.839928
*Borrelia americana*	Orange	*I*. *spinipalpis*	Nymph	12–0785	MH781137	33.573899, -117.839928
*Borrelia americana*	San Mateo	*I*. *pacificus*	Female	12–0040	KY563151	37.363597, -122.246426
*Borrelia americana*	Charleston, South Carolina	*I*. *minor*		Control	HM802226.1	
*Borrelia americana*	Charleston, South Carolina	*I*. *minor*		Control	EF015630.1	
*Borrelia bissettiae*	Marin	*I*. *pacificus*	Female	08–0745	KY563181	37.835500, -122.478300
*Borrelia bissettiae*	Orange	*I*. *spinipalpis*	Nymph	12–0780	KY563143	33.573899, -117.839928
*Borrelia bissettiae*	Orange	*I*. *spinipalpis*	Female	12–0438	MH781138	33.573899, -117.839928
*Borrelia bissettiae*	Orange	*I*. *spinipalpis*	Nymph	12–0776	MH781139	33.573899, -117.839928
*Borrelia bissettiae*	Orange	*I*. *spinipalpis*	Nymph	12–0743	KY563141	33.573899, -117.839928
*Borrelia bissettiae*	Orange	*I*. *spinipalpis*	Nymph	12–0454	KY563140	33.573899, -117.839928
*Borrelia bissettiae*	Orange	*I*. *spinipalpis*	Nymph	12–0451	MH781140	33.573899, -117.839928
*Borrelia bissettiae*	Orange	*I*. *spinipalpis*	Nymph	12–0755	KY563142	33.573899, -117.839928
*Borrelia bissettiae*	San Mateo	*I*. *pacificus*	Female	13–1009	KY563199	37.390048, -122.257426
*Borrelia bissettiae*	San Mateo	*I*. *pacificus*	Female	13–1040	KY563201	37.390048, -122.257426
*Borrelia bissettiae*	Santa Cruz	*I*. *pacificus*	Nymph	11–1420	KY563139	37.014408, -122.084290
*Borrelia bissettiae*	Contra Costa	*I*. *pacificus*		CA389—Control	NR148750.1	
*Borrelia burgdorferi ss*	Butte	*I*. *pacificus*	Nymph	11–1646	KY563178	39.52121, -121.44802
*Borrelia burgdorferi ss*	Butte	*I*. *pacificus*	Nymph	11–1702	KY563179	39.52121, -121.44802
*Borrelia burgdorferi ss*	Butte	*I*. *pacificus*	Nymph	11–1735	KY563180	39.52121, -121.44802
*Borrelia burgdorferi ss*	Butte	*I*. *pacificus*	Female	12–1336	KY563153	39.52121, -121.44802
*Borrelia burgdorferi ss*	Calaveras	*I*. *pacificus*	Male	11–1827	KY563174	38.022044, -120.549008
*Borrelia burgdorferi ss*	Contra Costa	*I*. *pacificus*	Nymph	08–1104	KY563182	37.900529, -122.256376
*Borrelia burgdorferi ss*	El Dorado	*I*. *pacificus*	Nymph	09–0752	KY563185	38.801096, -120.893394
*Borrelia burgdorferi ss*	El Dorado	*I*. *pacificus*	Nymph	10–0518	KY563134	38.770251, -121.040021
*Borrelia burgdorferi ss*	El Dorado	*I*. *pacificus*	Female	10–0556	KY563135	38.770251, -121.040021
*Borrelia burgdorferi ss*	El Dorado	*I*. *pacificus*	Male	11–0460	KY563159	38.770251, -121.040021
*Borrelia burgdorferi ss*	El Dorado	*I*. *pacificus*	Female	11–0480	KY563162	38.770251, -121.040021
*Borrelia burgdorferi ss*	El Dorado	*I*. *pacificus*	Female	11–0489	KY563163	38.770251, -121.040021
*Borrelia burgdorferi ss*	El Dorado	*I*. *pacificus*	Male	11–0570	KY563164	38.801096, -120.893394
*Borrelia burgdorferi ss*	Lake	*I*. *pacificus*	Female	09–0426	KY563183	38.910458, -122.592294
*Borrelia burgdorferi ss*	Lake	*I*. *pacificus*	Male	09–0671	KY563187	38.961918, -122.741246
*Borrelia burgdorferi ss*	Lake	*I*. *pacificus*	Nymph	11–1019	KY563168	39.017000, -122.813000
*Borrelia burgdorferi ss*	Lake	*I*. *pacificus*	Nymph	11–1073	KY563169	39.017000, -122.813000
*Borrelia burgdorferi ss*	Lake	*I*. *pacificus*	Nymph	13–0797	KY563191	39.262280, -122.950110
*Borrelia burgdorferi ss*	Lake	*I*. *pacificus*	Nymph	13–0800	KY563192	39.262280, -122.950110
*Borrelia burgdorferi ss*	Lake	*I*. *pacificus*	Nymph	13–0916	KY563194	39.262280, -122.950110
*Borrelia burgdorferi ss*	Lake	*I*. *pacificus*	Nymph	13–0937	KY563196	39.017000, -122.813000
*Borrelia burgdorferi ss*	Lake	*I*. *pacificus*	Nymph	13–0963	KY563198	39.017000, -122.813000
*Borrelia burgdorferi ss*	Marin	*I*. *pacificus*	Nymph	10–0106	KY563126	38.006443, -122.494629
*Borrelia burgdorferi ss*	Marin	*I*. *pacificus*	Nymph	10–0112	KY563127	38.006443, -122.494629
*Borrelia burgdorferi ss*	Marin	*I*. *pacificus*	Nymph	10–0113	MH781141	38.006443, -122.494629
*Borrelia burgdorferi ss*	Marin	*I*. *pacificus*	Nymph	10–0120	KY563128	38.006443, -122.494629
*Borrelia burgdorferi ss*	Marin	*I*. *pacificus*	Nymph	10–0682	KY563129	38.006443, -122.494629
*Borrelia burgdorferi ss*	Marin	*I*. *pacificus*	Nymph	10–0853	KY563136	38.006443, -122.494629
*Borrelia burgdorferi ss*	Marin	*I*. *pacificus*	Nymph	10–1031	MH781142	38.006443, -122.494629
*Borrelia burgdorferi ss*	Marin	*I*. *pacificus*	Nymph	10–1045	KY563130	38.006443, -122.494629
*Borrelia burgdorferi ss*	Marin	*I*. *pacificus*	Nymph	11–2405	KY563156	38.006443, -122.494629
*Borrelia burgdorferi ss*	Marin	*I*. *pacificus*	Nymph	11–2530	MH781143	38.006443, -122.494629
*Borrelia burgdorferi ss*	Marin	*I*. *pacificus*	Male	11–2554	KY563154	38.006443, -122.494629
*Borrelia burgdorferi ss*	Marin	*I*. *pacificus*	Female	12–0166	KY563149	38.006443, -122.494629
*Borrelia burgdorferi ss*	Marin	*I*. *pacificus*	Male	12–1134	KY563150	38.006443, -122.494629
*Borrelia burgdorferi ss*	Mariposa	*I*. *pacificus*	Male	10–1001	KY563171	37.292633, -120.147953
*Borrelia burgdorferi ss*	Nevada	*I*. *pacificus*	Female	10–0719	KY563170	39.330100, -120.986900
*Borrelia burgdorferi ss*	Sacramento	*I*. *pacificus*	Nymph	11–2083	KY563157	38.653200, -121.210900
*Borrelia burgdorferi ss*	San Luis Obispo	*I*. *pacificus*	Female	15–0797	MH781147	35.422511, -120.739472
*Borrelia burgdorferi ss*	San Mateo	*I*. *pacificus*	Female	A13-0477	KY563202	37.277536, -122.223845
*Borrelia burgdorferi ss*	San Mateo	*I*. *pacificus*	Male	12–0051	KY563148	37.363597, -122.246426
*Borrelia burgdorferi ss*	San Mateo	*I*. *pacificus*	Male	13–0194	KY563189	37.472000, -122.280000
*Borrelia burgdorferi ss*	Santa Clara	*I*. *pacificus*	Female	11–0326	KY563158	37.325300, -122.178900
*Borrelia burgdorferi ss*	Santa Clara	*I*. *pacificus*	Nymph	11–1358	KY563172	37.324000, -122.176000
*Borrelia burgdorferi ss*	Santa Clara	*I*. *pacificus*	Nymph	11–1673	KY563144	37.186126, -121.537900
*Borrelia burgdorferi ss*	Santa Clara	*I*. *pacificus*	Nymph	11–1685	KY563145	37.186126, -121.537900
*Borrelia burgdorferi ss*	Santa Clara	*I*. *pacificus*	Nymph	11–1686	MH781144	37.186126, -121.537900
*Borrelia burgdorferi ss*	Santa Clara	*I*. *pacificus*	Nymph	11–1688	KY563146	37.186126, -121.537900
*Borrelia burgdorferi ss*	Santa Clara	*I*. *pacificus*	Nymph	11–2111	KY563147	37.186126, -121.537900
*Borrelia burgdorferi ss*	Santa Clara	*I*. *pacificus*	Female	11–2322	KY563155	37.405632, -122.305901
*Borrelia burgdorferi ss*	Santa Clara	*I*. *pacificus*	Female	13–0855	KY563193	37.277758, -122.151275
*Borrelia burgdorferi ss*	Santa Cruz	*I*. *pacificus*	Female	09–0532	KY563184	37.014408, -122.084290
*Borrelia burgdorferi ss*	Santa Cruz	*I*. *pacificus*	Nymph	10–0146	KY563131	37.014408, -122.084290
*Borrelia burgdorferi ss*	Santa Cruz	*I*. *pacificus*	Nymph	10–0172	KY563132	37.014408, -122.084290
*Borrelia burgdorferi ss*	Santa Cruz	*I*. *pacificus*	Nymph	10–0201	KY563133	37.014408, -122.084290
*Borrelia burgdorferi ss*	Santa Cruz	*I*. *pacificus*	Nymph	11–1429	KY563175	37.014408, -122.084290
*Borrelia burgdorferi ss*	Santa Cruz	*I*. *pacificus*	Nymph	11–1514	KY563177	37.014408, -122.084290
*Borrelia burgdorferi ss*	Sonoma	*I*. *pacificus*	Nymph	09–0824	KY563186	38.343912, -122.547333
*Borrelia burgdorferi ss*	Sonoma	*I*. *pacificus*		CA4—Control	MH781145	
*Borrelia burgdorferi ss*	Sonoma	*I*. *pacificus*		CA8—Control	MH781146	
*Borrelia burgdorferi ss*	Westchester, New York	*I*. *scapularis*		N40—Control	CP002228.1	

## Discussion

This is the first study that characterizes the genetic diversity and large-scale geographic sub-structuring of *B*. *burgdorferi* sl over a large region of western North America. *Borrelia burgdorferi* sl includes *B*. *burgdorferi* ss, the agent of Lyme disease in North America, as well as other closely related spirochetes that have not yet been implicated as human pathogens, such as *B*. *bissettiae* and *B*. *americana*.

### *Borrelia burgdorferi* sensu stricto

Previous studies in western North America have highlighted northwestern California and the western slopes of the northern Sierra Nevada foothills as regions with moderate to high risk of exposure to the Lyme disease bacteria, *B*. *burgdorferi* ss. In northern California, *I*. *pacificus* nymphal tick infection prevalence average is 5% [12], but can be as high as 20 to 40% in some localities [24–26]. This prevalence is similar to many regions highly endemic for Lyme disease in the eastern and mid-western United States [27, 28]. Nevertheless, while *I*. *pacificus* ticks are found in many areas of western North America and present a risk of transmitting Lyme disease to people, this risk is not uniform throughout the region. For example, despite thousands of ticks tested to date, the only ticks found positive for *B*. *burgdorferi* ss from southern California are one adult *I*. *pacificus* and two *Dermacentor occidentalis* from Los Angeles County [29], and a single *Ixodes peromysci* nymph from Santa Barbara County [30]. Although *D*. *occidentalis* attaches to humans, it is not a competent vector of *B*. *burgdorferi* ss [31]. *Ixodes peromysci* is an uncommon tick that feeds predominately on *Peromyscus* spp. mice, and previously has been considered to be endemic only to the Channel Islands, off the coast of southern California [32]. To date, only a single *I*. *pacificus* has tested positive for *B*. *burgdorferi* ss from southern California. Our current findings further indicate that the acarological risk of acquiring Lyme disease in southern California is exceedingly low [29].

### Borrelia bissettiae

*Borrelia bissetti*ae (formerly *B*. *bissettii)* [6, 33] is a potential human pathogen in the United States and in Europe. In the Czech Republic, *B*. *bissettiae* was detected by PCR from sera of seven patients suspected to have Lyme borreliosis [8]. This spirochete was detected also by PCR from cardiac-valve tissue from a patient with endocarditis and aortic valve stenosis [7] and from a lymphocytomic breast tissue lesion from a Slovenian patient [9]. In the United States, *B*. *bissettiae* was detected by PCR from plasma cultured from a resident of southeastern North America [10]. In northwestern California, serum specimens from three residents of a rural community at high risk of tick-exposure and who were PCR positive for *B*. *burgdorferi* sl, were found to have been infected with *B*. *bissettiae* by sequence analysis [11]. However, none of those individuals had a clinical history compatible with Lyme disease [11, 34].

In this present study, *B*. *bissettiae* was detected in *I*. *pacificus* and *I*. *spinipalpis* adults and nymphs in coastal regions of both northern and southern California. This spirochete was first isolated from an adult *I*. *pacificus* from Del Norte County in the far north-coastal quadrant of California [33, 35]. Subsequently, it was detected in *I*. *pacificus* and *I*. *spinipalpis* in a few other regions of western North America [6, 36]. Recent studies have detected *B*. *bissettiae* in wild rodents and *Ixodes* spp. ticks in the midwestern and southeastern United States, Europe, and recently in South America [37–40]. Interestingly, *B*. *bissettiae* is recorded rarely from the northeastern United States, a region that harbors a remarkably high tick-infection prevalence with *B*. *burgdorferi* ss.

In California, *B*. *bissettiae* is found commonly in association with dusky-footed woodrats (*Neotoma fuscipes*), big-eared woodrats (*Neotoma macrotis*), Allen’s chipmunks (*Neotamias senex*), and *I*. *spinipalpis* [5, 36, 41, 42]. In addition, *B*. *bissettiae* has been detected in the bird tick *Ixodes auritulus* [14] and in sylvatic bird blood samples [43]. Genetic sub-structuring of Californian *B*. *burgdorferi* sl has been reported on a finer-scale within a single California county: *B*. *burgdorferi* ss was found in ticks from inland areas with higher than average temperatures whereas *B*. *bissettiae* was found in ticks from coastal areas with cooler temperatures [5]. These local regional differences in tick diversity may align with habitat types (e.g., chaparral, riparian, oak-woodland), which in turn can support different host species and potential reservoirs for different *Borrelia* genospecies [5].

### Borrelia americana

*Borrelia americana* was first isolated from *Ixodes minor* nymphs and birds in South Carolina as well as from *I*. *pacificus* from California [44]. Since then, it has been detected from ticks outside the United States, with recent detections in *Ixodes persulcatus* in China [45]. The pathogenic status of this spirochete is unclear but *B*. *americana*-like DNA reportedly has been amplified from patients with Lyme disease–like symptoms from the southern United States [46]. The first detection of *B*. *burgdorferi* sl in southern California was an isolate from an *I*. *pacificus* tick collected in Orange County [47], later named CA-29-91 [48], and ultimately renamed *B*. *americana* [44]. More recently, *B*. *americana* was detected in *I*. *pacificus* from Los Angeles and Alameda counties [5, 29]. In the current study, it is notable that *B*. *americana* was detected in both the north-coastal (San Mateo County) and south-coastal (Orange County) regions of the state, as well as in two tick species, e.g., an *I*. *pacificus* adult from San Mateo County and two *I*. *spinipalpis* nymphs from Orange County (Table 1).

### Borrelia miyamotoi

Other *Borrelia* spp. that cause human disease in North America include relapsing fever *Borrelia* that are molecularly and clinically distinct from *B*. *burgdorferi* sl infections. While most relapsing fever *Borrelia*, such as *B*. *hermsii*, are typically associated with argasid (soft) ticks in the genus *Ornithodoros*, *B*. *miyamotoi* is vectored by *Ixodes* species ticks in Europe, North America, and Asia. This spirochete recently was identified as an emerging pathogen in Russia, the Netherlands, Japan, and northeastern United States, and is associated with an acute febrile illness and subsequent relapsing fevers if left untreated [49]. Although no human cases of *B*. *miyamotoi* infection have been confirmed in California, serological, ecological, and epidemiological data offer presumptive evidence that *B*. *miyamotoi* occasionally infects people in northwestern California [50]. Molecular strain differences among *B*. *miyamotoi* appears to align with its associated tick species, with little geographic substructuring [51]. Similar to a 1% infection prevalence in other *Ixodes* ticks (both adults and nymphs) in surveillance conducted in the United States, Canada,and in Europe [15, 52, 53], *B*. *miyamotoi* is found in approximately 1% of *I*. *pacificus* nymphs and adults in California [12, 54]. While there is evidence of *B*. *miyamotoi* in rodents [15, 55], this similarity of infection prevalence among geographic regions, with diverse vectors and potential reservoir hosts, suggests a strong reliance on transovarial transmission to maintain infection in an area. Unlike *B*. *burgdorferi* sl, *B*. *miyamotoi* can be maintained transovarially and can be found in larval *I*. *pacificus* [12, 56]. In California, *B*. *miyamotoi* was detected primarily in *I*. *pacificus* from the northern region of the state, and was rarely detected in southern Californian *I*. *pacificus* [12]. To date, *B*. *miyamotoi* has not been found in *I*. *spinipalpis* nor any other wildlife tick in western North America.

## Conclusion

Our findings demonstrate large-scale geographic structuring of the *B*. *burgdorferi* sl complex in western North America with concomitant differential acarological risk of exposure to Lyme borreliosis spirochetes. In southern California, people are at an exceeding low acarological risk of exposure to *B*. *burgdorferi* ss, the agent of Lyme disease in North America. In this study, ticks infected with *B*. *burgdorferi* ss were found in the Sierra Nevada foothills, north coastal, and central coastal regions of California, as far south as San Luis Obispo County. The geographic distribution of *B*. *burgdorferi* ss in California coincides with epidemiological findings, with the highest incidence of Lyme disease reported in northern California [57]. Notably, only a single *I*. *pacificus* has tested positive for *B*. *burgdorferi* ss from southern California, despite decades of testing and thousands of ticks tested [CDPH, unpublished data; 21]. While the risk of acquiring Lyme disease may be low in southern California, the risk of exposure to other tick-borne pathogens, such as spotted-fever group rickettsia may be higher in this region of the state [58]. Public health education messages should highlight this differential risk of tick-borne diseases to health care providers and the public.

While people are not at acarological risk of exposure to *B*. *burgdorferi* ss in southern California, this study did find three other *Borrelia* in ticks from this region: *B*. *bissettiae*, *B*. *americana*, and *B*. *miyamotoi*. Likewise, the acarological risk of exposure to *B*. *bissettiae* is variable among California regions, with an evident association with coastal regions of the state, including coastal areas of southern California. Of note, no *I*. *pacificus* from the Sierra Nevada foothills were positive for *B*. *bissettiae*, while *B*. *burgdorferi* ss is found commonly in *I*. *pacificus* from that region.

This study provides an assessment of acarological risk for known human tick-borne disease pathogens as well as potentially novel human pathogenic *Borrelia* species over a broad geographic area. Prior understanding of regional risk of known and potential tick-borne disease agents can assist with advancing diagnostics and epidemiologic investigations of human tick-borne disease cases. Results from this study indicate that additional research is warranted to evaluate fine scale landscape or reservoir host distribution range and tick-borne disease prevalence in California.
